# Antimicrobial Evaluation of Diterpenes from *Copaifera langsdorffii* Oleoresin Against Periodontal Anaerobic Bacteria

**DOI:** 10.3390/molecules16119611

**Published:** 2011-11-18

**Authors:** Ariana B. Souza, Maria G. M. de Souza, Maísa A. Moreira, Monique R. Moreira, Niege A. J. C. Furtado, Carlos H. G. Martins, Jairo K. Bastos, Raquel A. dos Santos, Vladimir C. G. Heleno, Sergio Ricardo Ambrosio, Rodrigo C. S. Veneziani

**Affiliations:** 1 Nucleus of Research in Sciences and Technology, University of Franca, Franca, SP, 14404-600, Brazil; 2 Faculty of Pharmaceutical Sciences of Ribeirao Preto, University of Sao Paulo, Ribeirao Preto, SP, 14040-903, Brazil

**Keywords:** copalic acid, periodontitis, *Porphyromonas gingivalis*, copaiba oleoresin, *Copaifera langsdorffii*

## Abstract

The antimicrobial activity of four labdane-type diterpenes isolated from the oleoresin of *Copaifera langsdorffii* as well as of two commercially available diterpenes (sclareol and manool) was investigated against a representative panel of microorganisms responsible for periodontitis. Among all the evaluated compounds, (−)-copalic acid (CA) was the most active, displaying a very promising MIC value (3.1 µg mL^−1^; 10.2 µM) against the key pathogen (*Porphyromonas gingivalis*) involved in this infectious disease. Moreover, CA did not exhibit cytotoxicity when tested in human fibroblasts. Time-kill curve assays performed with CA against *P. gingivalis* revealed that this compound only inhibited the growth of the inoculums in the first 12 h (bacteriostatic effect). However, its bactericidal effect was clearly noted thereafter (between 12 and 24 h). It was also possible to verify an additive effect when CA and chlorhexidine dihydrochloride (CHD, positive control) were associated at their MBC values. The time curve profile resulting from this combination showed that this association needed only six hours for the bactericidal effect to be noted. In summary, CA has shown to be an important metabolite for the control of periodontal diseases. Moreover, the use of standardized extracts based on copaiba oleoresin with high CA contents can be an important strategy in the development of novel oral care products.

## 1. Introduction

Periodontitis is characterized by local infection and inflammation in tooth-supporting tissues and, if untreated, can result in tooth loss [1]. Moreover, many several systemic infections (bacteraemia, endocarditis, brain abscesses, and urogenital, skin and soft tissue, pulmonary, and gastrointestinal infections) are also associated with periodontal anaerobic pathogens. The different susceptibilities of these pathogens to antimicrobial agents makes therapy very difficult [2], thus denoting the urgent need for discovering novel and safe compounds for application in the control of this disease.

According to several authors, natural products have been a rich and promising source for the discovery of novel biologically active compounds [3]. Among all classes of plant metabolites, diterpenes are considered to display a wide spectrum of biological activities, including antibacterial activity [4,5,6,7]. Searches in the scientific literature using the PubMed and Scifinder databases have demonstrated that several classes of diterpenoids, such as pimarane, clerodane, kaurane, isopimarane, labdane and others can be considered as a potential source of antimicrobial agents [8,9,10,11,12,13].

Recently, our research group has demonstrated that some pimarane diterpenes are able to inhibit the growth of the main microorganisms responsible for periodontal diseases with very promising minimal inhibitory concentration (MIC) values [14] These results have allowed us to conclude that this class of natural compounds is an important source for the discovery of new efficient bioactive metabolites for the control of periodontitis.

In agreement with our early findings and as part of our ongoing efforts to explore the antimicrobial potential of diterpenes against the pathogens responsible for periodontal diseases, our research group has decided to investigate the activity of a different class of diterpenes. For this purpose, a commercially available and well documented source of *ent*-labdane-type diterpenes [15], namely the oleoresin of *Copaifera langsdorffii*, was chosen. Furthermore, an antimicrobial evaluation was also carried out with two other commercially available labdane-type diterpenes of normal series (manool and sclareol), in order to obtain a wider view of such class of metabolites against a representative panel of periodontal anaerobic pathogens.

## 2. Results and Discussion

**Figure 1:**
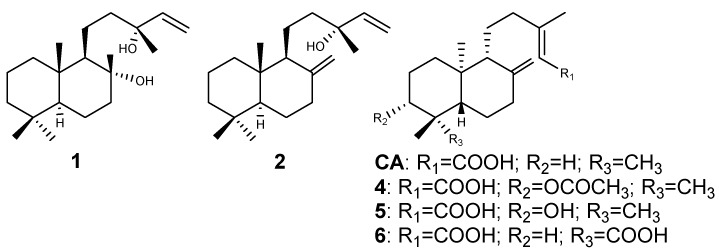
Chemical structures of the evaluated compounds. **1**: sclareol, **2**: manool, **CA**: (−)-copalic acid (CA), **4**: (−)-acetoxycopalic acid, **5**: (−)-hydroxycopalic acid, **6**: (−)-agathic acid.

The present work describes the isolation of four diterpenes from the oleoresin of *C. langsdorffii*. Their spectral data are in agreement with those previously reported in the literature: (−)-copalic acid (CA) [16], (−)-acetoxycopalic acid (**4**) [17], (−)-hydroxycopalic acid (**5**) [18], and (−)-agathic acid (**6)** [19]. The ^1^H- and ^13^C-NMR spectral data also indicated that the purity of each isolated compound lies between 95–98%. Figure 1 shows the structures of the identified metabolites as well as of the purchased diterpenes included in the antimicrobial evaluations sclareol and manool (**1** and **2**, respectively).

The antimicrobial evaluation of the diterpenes against the main periodontal anaerobic bacteria was performed by determining their MIC values by means of the microdilution broth method [20]. The obtained MIC values are listed in Table 1.

Concerning the antimicrobial assays of compounds isolated from natural sources, some authors [21,22] have established MIC value criteria for determination of their antimicrobial potential. These authors suggested that MIC values higher than 100.0 µg mL^−1^ for pure metabolites are evidence of poor activity. On the other hand, isolated compounds that inhibit the growth of the microorganisms at concentrations below 10.0 µg mL^−1^ are considered very promising in the search for new anti-infection agents. Analyzing the results presented in Table 1, it can be observed that compounds **1**, **2**, and **CA** are the most active, since they furnished very promising MIC values for several tested bacteria that are closely associated with periondontitis.

Antimicrobial compounds have demonstrated different action mechanisms [23]. Several authors have emphasized that diterpenes are an important class of plant metabolites for the search of new antibacterial agents; however, the mechanism(s) responsible for this property have not yet been very well elucidated [24]. Urzúa *et al.* [24] have suggested that these metabolites promote bacterial lysis and disruption of the cell membrane. According to these authors, the structural features that promote the efficient antibacterial activity include a lipophilic structure, capable of insertion into the cell membrane, and one strategically positioned hydrogen-bond-donor group (HBD; hydrophilic group), which interacts with the phosphorylated groups on the membrane. In these studies, it was also emphasized that a second HBD introduced in the lipophilic region led to reduction in or suppression of the activity. A careful observation of the results depicted in Table 1 reveals that compound **2** and **CA**, which contains only one HBD, displays much lower MIC values than those achieved with diterpenes **4**–**6**, which contain two HBD in their structures. Based on these considerations, our results give support to the mechanism of action suggested by Urzúa *et al.* [24]. However, the significant MIC values displayed by **1** (Table 1) against some microorganisms reveals that the antibacterial activity displayed by this class of natural products is also ruled by other structural features. These statements justify the need for more information about the anti-periodontitis potential of a large number of diterpenes, so that further quantitative structure-active relationship (QSAR) studies can be accomplished. Among all the obligate anaerobic bacteria related to this infection, *Porphyromonas gingivalis* can be considered one of the most important since it produces several endodontopathogenic materials that suppress host defense mechanisms and destroy periodontal tissues [2,25,26]. When considering the MIC values at the micromolar level, it is possible to observe that CHD (1.6 µM) is about six times more potent than CA (10.2 µM). Nevertheless, several adverse effects are associated with the regular use of CHD [7,27]. This reinforces the great importance of CA as a prototype for the development of novel and safe bioactive compounds for the control of periodontitis.

The literature contains descriptions of some natural compounds that are very active against *P. gingivalis*. Park *et al*. [25] have reported that (10)-gingerol, a natural product isolated from the rhizomes of ginger, is able to inhibit the growth of *P. gingivalis* with an MIC value of 6.0 µg mL^−1^ (17.2 µM), which was considered a potent *in vitro* activity. Against the same microorganism, Katsura *et al*. [28] have demonstrated a very promising MIC value of 4.0 µg mL^−1^ (15.6 µM) for bakuchiol, a metabolite isolated from the seeds of *Psoralea corylifolia*. However, these authors also mentioned that this compound was cytotoxic against L929 cells in cell culture.

Bearing all these data in mind, it is possible to observe that CA is almost one and a half time more potent than (10)-gingerol and bakuchiol against this important periodontal pathogen. Unlike bakuchiol, (−)-copalic acid is not cytotoxic against human fibroblasts in the range 7.8 to 62.5 µM. These results confirm the role of CA as an important and selective metabolite that should be considered for the control of periodontitis.

**Figure 2:**
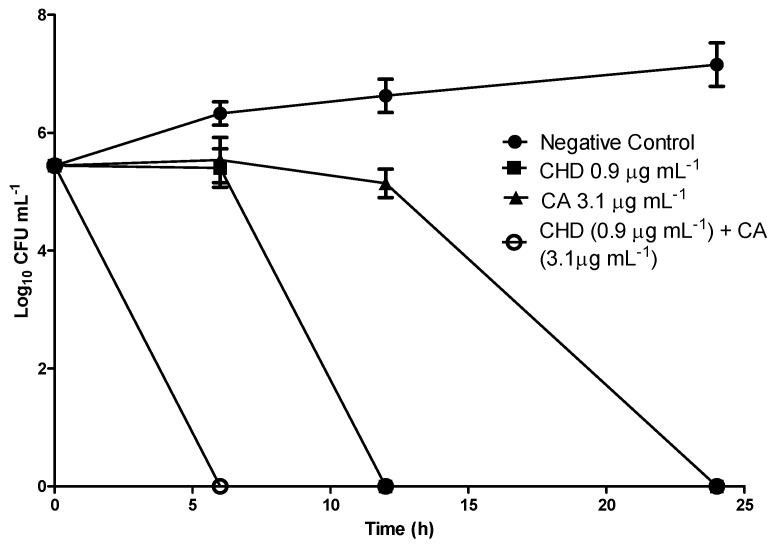
Time-kill curves for CA, CHD, and CHD + CA against *P. gingivalis*.

The interesting results obtained for CA against the main pathogen related to periodontitis motivated us to conduct time-kill curve assays against *P. gingivalis* (5 × 10^5^ CFU mL^−1^) using this compound at 3.1, 6.2, and 12.4 µg mL^−1^ (respectively one, two, and three times its MBC). CHD was used as positive control at its MBC value (0.9 µg mL^−1^). The constructed curves are depicted in Figure 2.

CA at 3.1 µg mL^−1^ is able to completely kill *P. gingivalis* after only 24 h of incubation. Analysis of these data also suggests that CA only inhibits the growth of the inoculum in the first 12 h. This indicates that CA displays a bacteriostatic effect during this period, but its bactericidal effect is clearly noted thereafter (between 12 and 24 h). There were no statistical differences among the periods of time investigated for each concentration, allowing us to conclude that there are no dose-dependent responses for this compound in the conditions employed during the assays. Therefore, only the smallest CA concentration of (3.1 µg mL^−1^) is presented in Figure 2. It is also possible to verify the existence of an additive effect when CA and CHD are associated at their MBC values. The time curve profile resulting from this combination reveals that only six hours is necessary for this association to exhibit its bactericidal effect against *P. gingivalis*. This is a remarkable result, considering the relatively short time period that oral care active ingredients remain in the oral cavity.

## 3. Experimental Section

### 3.1. General

NMR spectra were recorded on a Bruker Avance DRX 400 spectrometer (400 MHz for ^1^H and 100 MHz for ^13^C). Samples were dissolved in CDCl_3_, and TMS was used as internal reference. Authentic oleoresin from *Copaifera langdsdorffii* was kindly provided by the Brazilian company Apis-Flora Comercial e Industrial. The commercially available sclareol (**1**) and manool (**2**) were purchased from Glycosyn and Sigma-Aldrich^®^, respectively.

### 3.2. Isolation of Compounds

About 12.0 g of oleoresin was re-fractioned using several chromatographic techniques, such as vacuum liquid chromatography, flash chromatography, and preparative thin layer chromatography, as previously described [15]. These procedures furnished the compounds (−)-copalic acid (CA, 210.0 mg), (−)-acetoxycopalic acid **(4**, 130.0 mg), (−)-hydroxycopalic acid (**5**, 60.0 mg), and (−)-agathic acid (**6**, 72.0 mg) that were identified through ^1^H and ^13^C NMR data comparison with the literature [16,17,18,19].

### 3.4. Determination of the Minimal Inhibitory Concentration and Minimal Bactericidal Concentration

The minimal inhibitory concentration values (MIC) of the obtained compounds were determined in triplicate by using the microdilution broth method in 96-well microplates [20]. The tested strains were obtained from the American Type Culture Collection (ATCC). The following microorganisms were assessed in the present work: *Porphyromonas gingivalis* (ATCC 33277), *Prevotella nigrescens* (ATCC 33563), *Fusobacterium nucleatum* (ATCC 25586), *Bacteroides fragilis* (ATCC 25285), *Actinomyces naeslundii* (ATCC 19039), *Bacteroides thetaiotaomicron* (ATCC 29741), and *Peptostreptococcus anaerobius* (ATCC 27337). The samples were dissolved in dimethyl sulfoxide (DMSO) at 1.0 mg mL^−1^, followed by dilution in Schadler broth (Difco, Kansas City, MO, USA) supplemented with hemin (5.0 µg mL^−1^) and vitamin K1 (10.0 µg mL^−1^); concentrations ranging from 0.2 to 400.0 µg mL^−1^ were achieved. The final DMSO content was 5% (v/v), and this solution was used as negative control. The inoculum was adjusted for each organism, to yield a cell concentration of 5 × 10^5^ colony forming units (CFU) mL^−1^, according to a previous standardization by the Clinical Laboratory Standards Institute [20]. One inoculated well was included, to enable control of the adequacy of the broth for organism growth. One non-inoculated well, free of antimicrobial agent, was also utilized, to ensure medium sterility. Chlorhexidine dihydrochloride (CHD) was used as positive control. The microplates (96-wells) were sealed with plastic film. The time necessary for growth was 72 h and incubation was accomplished at 37 °C in an anaerobic work station (Don Whitley Scientific, Bradford, UK), in 5–10% H_2_, 10% CO_2_, 80–85% N_2_ atmosphere. After that, resazurin (30 µL) in aqueous solution (0.02%) was added to the microplates, to indicate microorganism viability [29].

The minimal bactericidal concentration (MBC) was determined for **CA**. The MIC procedure was repeated for **CA** and *P. gingivalis*, and an aliquot of the inoculum was aseptically removed from the well presenting no apparent growth before the addition of resazurin, and then plated onto agar Schadler (Difco) supplemented with hemin (5.0 µg mL^−1^), vitamin K1 (10.0 µg mL^−1^), and sheep blood (5%); the plates were incubated as previously described.

### 3.5. Time-Kill Curves

Time-kill assays against *Porphyromonas gingivalis* were performed in triplicate based on D’arrigo *et al.* [30] and the average values were used to plot the graphs. Tubes containing **CA** at final concentrations of 3.1, 6.2, and 12.4 µg mL^−1^ (respectively one, two, and three times the MBC of **CA** for *P. gingivalis*) were inoculated with the target microorganism, resulting in a starting bacterial density of 5 × 10^5^ CFU mL^−1^, followed by incubation at 37 °C in the same previously described anaerobic conditions. Samples were removed for the determination of viable strains at 6, 12 and 24 h of incubation, followed by dilution, when necessary, in sterile fresh medium. The diluted samples (50 µL) were spread onto agar Schadler (Difco) supplemented with hemin (5.0 µg mL^−1^), vitamin K1 (10.0 µg mL^−1^), and sheep blood (5%), incubated at 37 °C, and counted after 48 h. Time-kill curves were constructed by plotting the log_10_ CFU mL^−1^ versus time. CHD at its MBC (0.9 µg mL^−1^) and a suspension of *P. gingivalis* without added CA were used as positive and negative control, respectively.

In a second set of experiments, which aimed to investigate the effect of the combination of **CA** with CHD on the time necessary for complete elimination of *P. gingivalis*, a time-kill curve was built for these chemicals. CHD and **CA** were used at their MBC values, and the same protocol described above was employed in this assay.

### 3.6. Cytotoxicity Assay

The effect of **CA** on cell viability was assessed by the XTT assay using the primary human fibroblast cell line obtained from Coriell Cell Repositories (Camden, NJ, USA). Briefly, cells were trypsinized and seeded in 96-well plate at a concentration of 10^4^ cells/well in DEMEM plus HAM-F10 (1:1, v/v) medium (Sigma, St. Louis, MO, USA) supplemented with 20% fetal bovine serum (Life Technologies, Carlsbad, CA, USA). After 24 h of incubation at 37 °C, cell cultures were treated with different concentrations of copalic acid dissolved in DMSO (1%), namely from 7.8 to 62.5 μM, for another 24 h. Then, cell viability was assessed with the Cell Proliferation Kit II (Roche, Mannheim, Germany) according to the manufacturer’s instruction. The absorbance of the orange formazan product was detected at 490 nm with the reference wavelength at 620 nm in a microplate reader Sunrise (Tecan, Männdorf, Switzerland). Cell viability was expressed as the percentage of the negative control. Doxorubicin at 3.0 µg mL^−1^ was used as positive control.

## 4. Conclusions

In conclusion, copalic acid is an important metabolite and should be considered for the control of periodontal diseases. Moreover, in previous work our research group has demonstrated that this diterpene is also very active against *Streptococcus mutans*, another key oral pathogen associated with dental caries. Therefore, these data denote that future oral care products containing this compound could be of great value for application in the treatment of control oral pathologies.

The oleoresin of copaiba, the natural product utilized in this study, is extensively commercialized in Brazil as a crude oil and in several pharmaceutical and cosmetic products, such as capsules, shampoos, soaps, capillary lotions, and bathing foams. Considering that (−)-copalic acid is the single diterpene that is always found in this botanical source, it is possible to conclude that the use of standardized extracts based on the oleoresin of copaiba with high CA contents is an important strategy in the development of novel oral care products.

## Figures and Tables

**Table 1:** *In vitro* antibacterial actitivity (MIC) of the tested diterpenes against periodontal bacteria.

Microorganism (ATCC)	Minimum inhibitory concentration in µg mL^−1^ [µM]						
	^#^ CHD	1	2	CA	4	5	6
*B. fragilis* (25285)	7.4 [12.8]	*	25.0 [86.1]	25.0 [82.1]	100.0 [275.9]	400.0 [1248.3]	400.0 [1196.0]
*A. naeslundii* (19039)	1.8 [3.1]	12.5 [40.8]	25.0 [86.1]	6.2 [20.4]	25.0 [69.0]	100.0 [312.0]	50.0 [149.5]
*P. gingivalis* (33277)	0.9 [1.6]	6.2 [20.2]	6.2 [21.3]	3.1 [10.2]	25.0 [69.0]	25.0 [78.0]	50.0 [149.5]
*P. nigrescens* (33563)	0.9 [1.6]	400.0 [1304.9]	12.5 [43.0]	200.0 [656.9]	200.0 [551.7]	200.0 [624.1]	200.0 [598.0]
*F. nucleatum* (25586)	1.8 [3.1]	400.0 [1304.9]	50.0 [172.1]	200.0 [656.9]	200.0 [551.7]	200.0 [624.1]	200.0 [598.0]
*B. thetaiotaomicron* (29741)	29.0 [50.1]	*	50.0 [172.1]	*	400.0 [1103.4]	400.0 [1248.2]	*
*P. anaerobius* (27337)	7.4 [12.8]	3.1 [10.1]	12.5 [43.0]	3.1 [10.2]	12.5 [34.5]	25.0 [78.0]	25.0 [74.7]

^#^ Positive control; * Inactive in the evaluated concentrations.
